# Supplementary material for: Solvation of Large Polycyclic Aromatic Hydrocarbons in Helium: Cationic and Anionic Hexabenzocoronene
Source: Molecules. 2022 Oct 10;27(19):6764. doi: 10.3390/molecules27196764 (PMC9573446; doi:10.3390/molecules27196764)
Supplement: Supplementary file 1 [file molecules-27-06764-s001.zip › molecules-1944992-supplementary.pdf]

Supplementary Material

Adsorption of Helium on large, planar polycyclic aromatic hydrocarbons: Cationic and anionic hexabenzocoronene

Miriam Kappe[1], Florent Calvo[2*], Johannes Schöntag[3], Holger Bettinger[3], Serge Krasnokutski[4], Martin Kuhn[1], Elisabeth Gruber[1], Fabio Zappa[1], Paul Scheier[1], Olof Echt[1,5*]

1 Institut für Ionenphysik und Angewandte Physik, Universität Innsbruck, 6020 Innsbruck, Austria
2 Université Grenoble Alpes, CNRS, LiPhy, 38000 Grenoble, France
3 Institut für Organische Chemie, Universität Tübingen, 72076 Tübingen, Germany
4 Laboratory Astrophysics and Cluster Physics Group of the MPI for Astronomy at the University of Jena, Helmholtzweg 3, D-07743 Jena, Germany
5 Department of Physics, University of New Hampshire, Durham NH 03824, USA
* Corresponding authors:
florent.calvo@univ-grenoble-alpes.fr (Florent Calvo), olof.echt@unh.edu (Olof Echt)

Data provided in this file (HBC_SI_220901f.docx):

- Fig. S1. Mass spectra of positively and negatively charged $Hbc^{\pm}$ ions complexed with helium.
- Fig. S2. First derivatives of logarithmic ion abundances for cation and anions, and second derivatives of computed energies.
- Fig. S3. Computed front and side views of He_nHbc^+ for $n \leq 90$. For each size, the helium densities obtained from the PIMD simulations are superimposed on the structure of Hbc^+.
- Table S1. Computed classical and quantum energies *versus* size n for $He_nHbc^{\pm}$.
- Table S2. Cartesian coordinates (x,y,z) and partial charges (q) on each atom of bare Hbc^+ computed using DFT at the M06-2X/6-31+G* level.
- Table S3. Cartesian coordinates (x,y,z) and partial charges (q) on each atom of bare Hbc^- computed using DFT at the M06-2X/6-31+G* level.

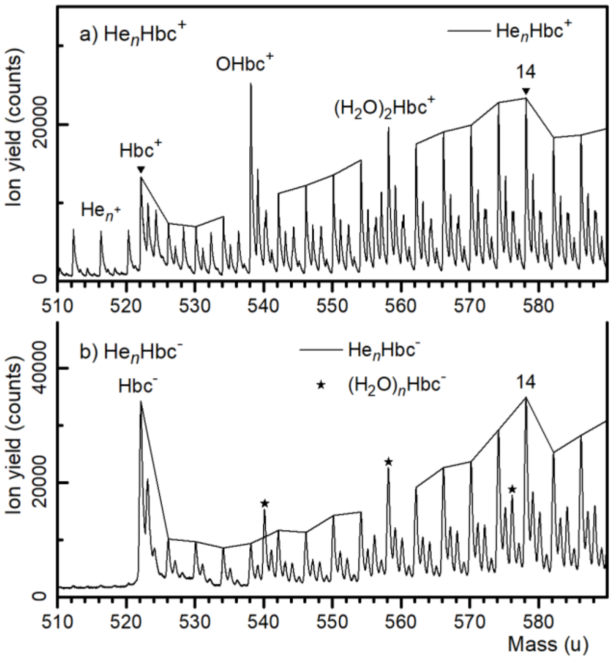

Figure. S1. Mass spectra of positively and negatively charged $Hbc^{\pm}$ ions complexed with helium (panels and b, respectively). Mass peaks due to isotopically pure $He_nHbc^{\pm}$ (i.e. $^4He_n{}^{12}C_{42}{}^1H_{18}$, mass 522.141 u for $n = 0$) are connected by solid lines. Contributions due to ^{16}O and $^1H_2{}^{16}O$ contaminants are labeled. In agreement with mass spectra obtained by laser-induced desorption [1], there is no evidence for the "fairly facile loss of up to four hydrogen atoms" from Hbc cations reported by Reed and Tennent upon electron ionization of bare Hbc [2].

1. Mavrinskaya, N.; Räder, H. J.; Müllen, K. Nonlinear behavior during semi-quantitative analysis of thin organic layers by laser desorption mass spectrometry. *Rapid Commun. Mass Spectrom.* **2011**, *25*, 2196-2200, doi:10.1002/rcm.5102.
2. Reed, R. I.; Tennent, A. Mass spectrum of 1-12, 2-3, 4-5, 6-7, 8-9, 10-11-hexabenzocoronene. *Org. Mass Spectrom.* **1971**, *5*, 619-621, doi:10.1002/oms.1210050513.

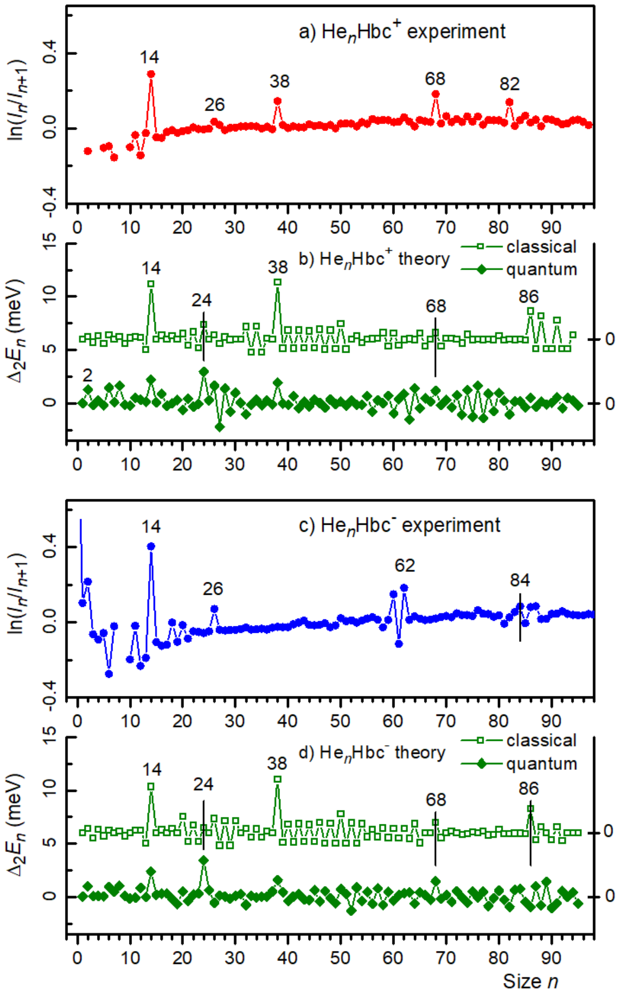

Figure. S2. Panels a and c: First derivatives of logarithmic ion abundances for cation and anions, respectively. The corresponding second derivatives of computed energies are displayed in panels b and d, respectively.

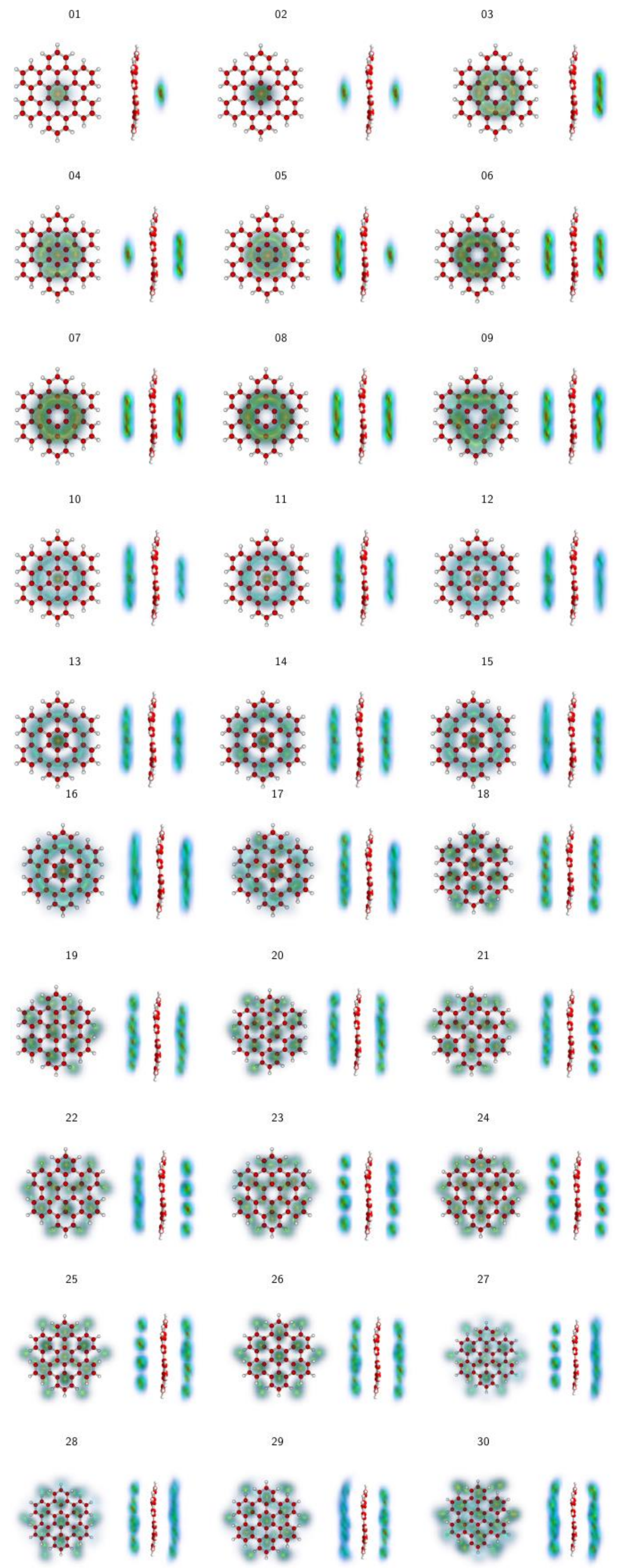
01
02
03
04
05
06
07
08
09
10
11
12
13
14
15
16
17
18
19
20
21
22
23
24
25
26
27
28
29
30

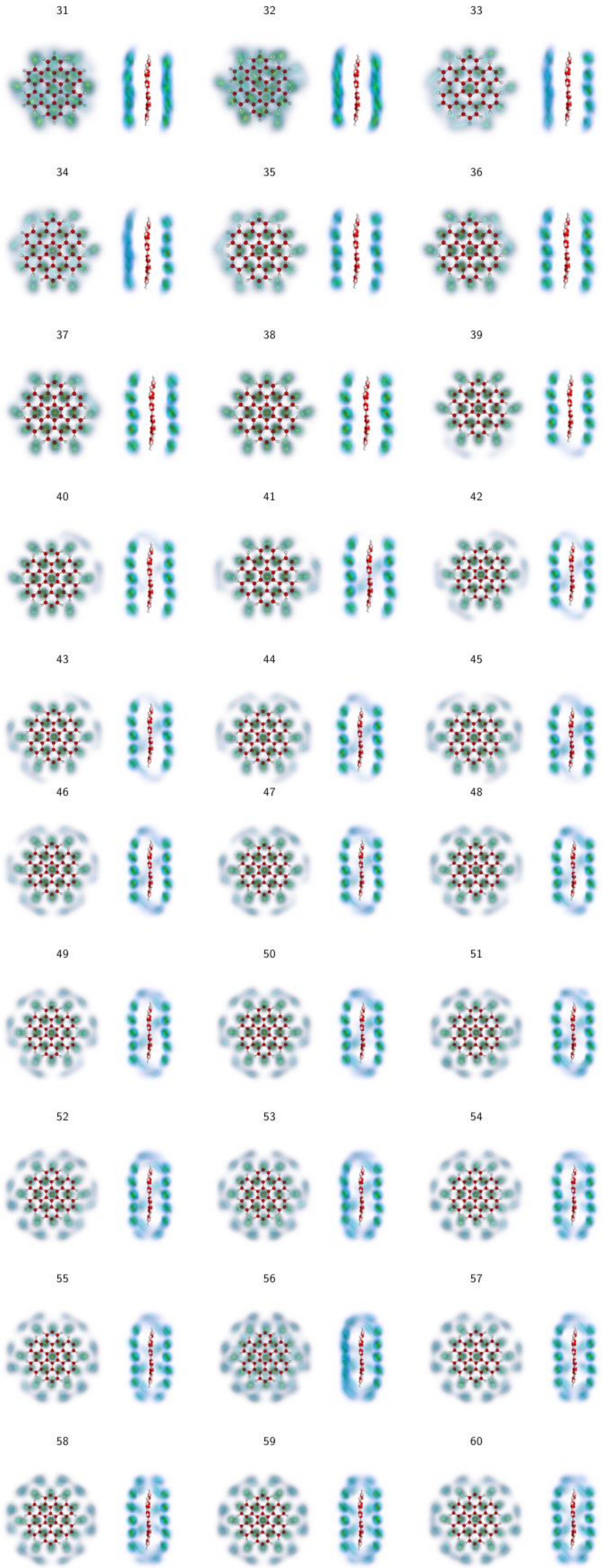
31
32
33
34
35
36
37
38
39
40
41
42
43
44
45
46
47
48
49
50
51
52
53
54
55
56
57
58
59
60

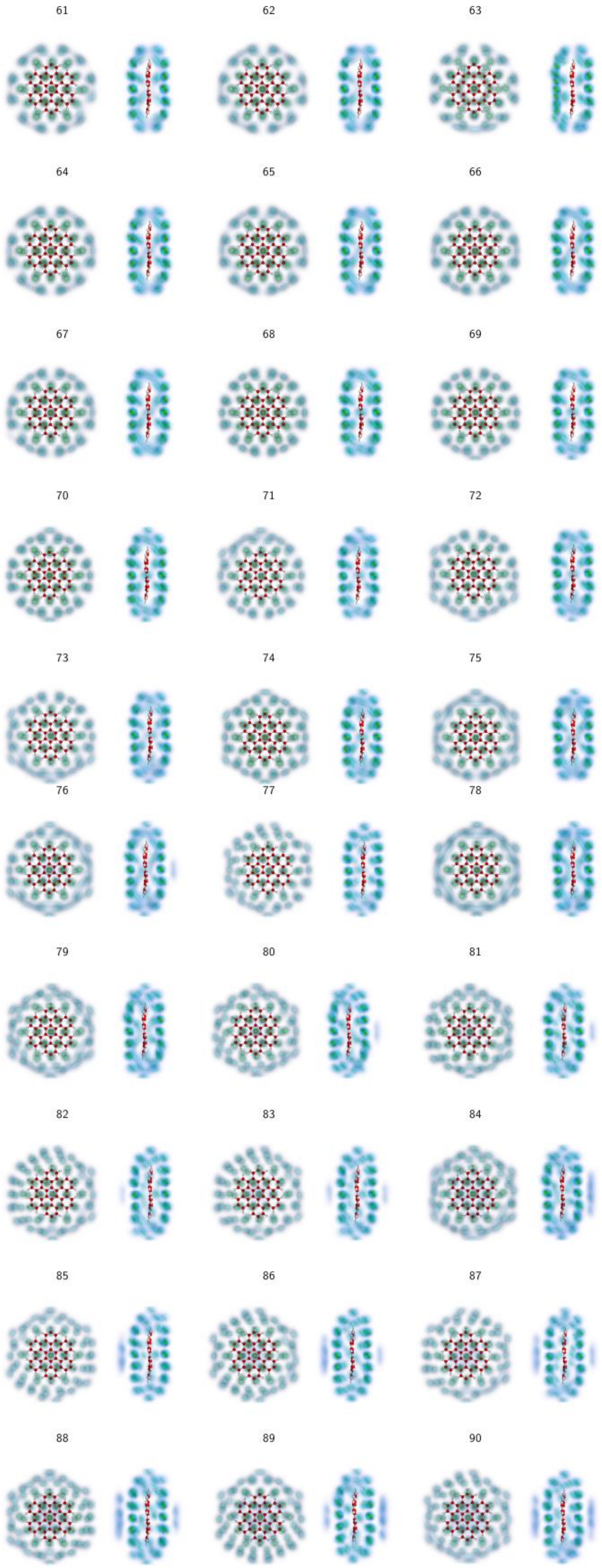

Figure. S3. Computed front and side views of He_nHbc^+ for $n < 90$. For each size, the helium densities obtained from the PIMD simulations are superimposed on the structure of Hbc^+.

Table S1. Computed energies *versus* size n for He_nHbc^+ and He_nHbc^-. Classical energies E_c are listed in meV per atom; total quantum energies E_q are listed in Hartree. The number of He atoms on either side of $Hbc^{\pm}$ is indicated for some sizes n as $p+q$, with $p+q = n$.

He_nHbc^+

n	E_c/n (meV)	arrangement	E_q (Hartree)	arrangement
0	0	0+0	0	
01	24.18947	1+0	-6.819429100869068E-004	
02	24.19581	1+1	-1.363778672277615E-003	
03	24.11157	3+0	-1.998802168255109E-003	
04	24.13835	3+1	-2.640218261895236E-003	
05	24.08661	4+1	-3.272270324304337E-003	
06	24.12241	3+3	-3.912133948980715E-003	
07	24.09351	4+3	-4.497461412557004E-003	
08	24.07104	4+4	-5.079374470285785E-003	
09	24.02515	5+4	-5.600732300559383E-003	
10	24.02920	7+3	-6.127710283877961E-003	6+4
11	24.02170	7+4	-6.661496498089828E-003	
12	23.99329	7+5	-7.176870273546458E-003	
13	23.95650	7+6	-7.680213892810647E-003	
14	23.99517	7+7	-8.178316826467170E-003	
15	23.68208	8+7	-8.594846555222033E-003	
16	23.40806	8+8	-9.007970570167983E-003	
17	23.14412	9+8	-9.388366269025264E-003	
18	22.90958	9+9	-9.777444754939824E-003	
19	22.68241	10+9	-1.016709739443137E-002	
20	22.47793	10+10	-1.054526561792713E-002	
21	22.26732	12+9	-1.094660765538418E-002	
22	22.10127	12+10	-1.133218722458680E-002	
23	21.91985	12+11	-1.172992129189334E-002	
24	21.78641	12+12	-1.212923421536824E-002	
25	21.60853	13+12	-1.242021590341250E-002	
26	21.44460	13+13	-1.270098125667590E-002	
27	21.27876	15+12	-1.292155947695439E-002	
28	21.13848	15+13	-1.322285608835159E-002	
29	21.00088	16+13	-1.347309807775598E-002	
30	20.87340	15+15	-1.375225445625423E-002	
31	20.75339	16+15	-1.399631084894553E-002	
32	20.64117	16+16	-1.423801823902286E-002	
33	20.49945	17+16	-1.451829660747848E-002	
34	20.40148	18+16	-1.480433530552930E-002	
35	20.27480	18+17	-1.507808802768300E-002	
36	20.18857	18+18	-1.535869692998750E-002	
37	20.10423	19+18	-1.562981070083166E-002	
38	20.02457	19+19	-1.590588835335648E-002	
39	19.81153		-1.611094388771822E-002	
40	19.63046		-1.631686923352261E-002	
41	19.43745		-1.652772420447603E-002	
42	19.27381		-1.671359438330699E-002	
43	19.09801		-1.691731672340760E-002	
44	18.94805		-1.711576472219008E-002	
45	18.78734		-1.732388974104613E-002	
46	18.65057		-1.751956933468870E-002	
47	18.49989		-1.771429968054831E-002	
48	18.37484		-1.792383864483180E-002	
49	18.23722		-1.812082086013770E-002	
50	18.12235		-1.832024239610316E-002	
51	17.98294		-1.851379333162801E-002	
52	17.86687		-1.871593138730249E-002	
53	17.75542		-1.890889066831103E-002	
54	17.64200		-1.910562087637529E-002	
55	17.53831		-1.930730725308037E-002	
56	17.43836		-1.948405924848980E-002	

57	17.34131	-1.968955056717587E-002
58	17.24617	-1.988490516138827E-002
59	17.14384	-2.007946441713342E-002
60	17.05585	-2.024880132826827E-002
61	16.96111	-2.045282868075907E-002
62	16.87862	-2.064245285043046E-002
63	16.79955	-2.079971461014480E-002
64	16.72230	-2.101268969669519E-002
65	16.64776	-2.117387344673725E-002
66	16.56668	-2.135224838897270E-002
67	16.49722	-2.151313499707107E-002
68	16.42956	-2.166816802953964E-002
69	16.35509	-2.178038991420668E-002
70	16.29173	-2.189915446309851E-002
71	16.22874	-2.200654339744468E-002
72	16.16664	-2.212775329575125E-002
73	16.10601	-2.222127109581134E-002
74	16.05193	-2.235404844442194E-002
75	15.99341	-2.244169419449153E-002
76	15.93685	-2.257457450532116E-002
77	15.88189	-2.264680789840260E-002
78	15.82891	-2.277010219368248E-002
79	15.77722	-2.286056622424507E-002
80	15.72713	-2.297763900433406E-002
81	15.67398	-2.305957748686307E-002
82	15.62308	-2.313263927907334E-002
83	15.57345	-2.324467600953441E-002
84	15.52542	-2.335066321096539E-002
85	15.47864	-2.345006016868611E-002
86	15.43367	-2.356367943430589E-002
87	15.35938	-2.365896120213054E-002
88	15.29695	-2.376677654177332E-002
89	15.21125	-2.386909272527251E-002
90	15.13719	-2.397523672418906E-002
91	15.07454	-2.408023571016252E-002
92	14.99417	-2.416516938158190E-002
93	14.92478	-2.426665934072722E-002
94	14.86647	-2.435018307739516E-002
95	14.80525	-2.442364401995693E-002
96	14.74498	-2.450390740524350E-002
97	14.67958	-2.457857187888973E-002
98	14.62306	-2.465169939497332E-002
99	14.56668	-2.472794201027822E-002
100	14.51121	-2.480685352856155E-002
101	14.45074	-2.487051631556957E-002
102	14.39842	-2.494968743716937E-002
103	14.34584	-2.502612924413018E-002
104	14.29408	-2.509003184761485E-002
105	14.24401	-2.518643102847318E-002
106	14.19422	-2.524355803577767E-002
107	14.14439	-2.530135509026720E-002
108	14.08999	-2.538342302369289E-002
109	14.03484	-2.544163331445132E-002
110	13.97784	-2.549908934545182E-002

He$_n$Hbc$^-$

n	E_c/n (meV)	arrangement	E_q (Hartree)	arrangement
01	25.12082	1+0	-7.116418775573115E-004	
02	25.12654	1+1	-1.420823471528046E-003	
03	24.98347	3+0	-2.092734140784047E-003	
04	25.02482	3+1	-2.761061024997960E-003	
05	24.97763	4+1	-3.425169514403443E-003	
06	24.99146	3+3	-4.086675045542022E-003	

07	24.96376	4+3	-4.713170824590654E-003	
08	24.93732	4+4	-5.320962083349337E-003	
09	24.89609	5+4	-5.889301144537721E-003	
10	24.88995	7+3	-6.452584862180117E-003	6+4
11	24.88397	7+4	-7.021205408035449E-003	
12	24.85830	7+5	-7.592436840325503E-003	
13	24.81747	7+6	-8.131017591997584E-003	
14	24.85175	7+7	-8.668839725736503E-003	
15	24.59070	8+7	-9.118123245984990E-003	
16	24.36182	8+8	-9.559279636643185E-003	
17	24.14161	9+8	-9.988352733290639E-003	
18	23.94585	9+9	-1.040492682996538E-002	
19	23.74842	10+9	-1.082741184497784E-002	
20	23.56941	10+10	-1.127319321144245E-002	
21	23.33293	11+10	-1.169906479332637E-002	
22	23.15235	12+10	-1.213826256198835E-002	
23	22.95443	12+11	-1.256896080431156E-002	
24	22.80534	12+12	-1.298700397331822E-002	
25	22.64714	13+12	-1.327810897679332E-002	
26	22.50022	13+13	-1.354490643642256E-002	
27	22.31444	14+13	-1.383051108387034E-002	
28	22.18341	15+13	-1.411122453405846E-002	
29	22.02168	15+14	-1.438980830387840E-002	
30	21.90924	15+15	-1.467417803147202E-002	
31	21.76729	16+15	-1.495397979838546E-002	
32	21.63409	16+16	-1.522273530177608E-002	
33	21.49686	17+16	-1.551794644929291E-002	
34	21.37936	18+16	-1.580781118422390E-002	
35	21.25754	18+17	-1.609886636420984E-002	
36	21.15299	18+18	-1.638847259402568E-002	
37	21.04948	19+18	-1.667607376498591E-002	
38	20.95144	19+19	-1.694406147064903E-002	
39	20.72935		-1.715319494178007E-002	
40	20.53933		-1.734537116521886E-002	
41	20.33814		-1.755044758327909E-002	
42	20.16636		-1.775295228073760E-002	
43	19.98256		-1.794600510916209E-002	
44	19.82473		-1.814792466474686E-002	
45	19.65671		-1.835874947886416E-002	
46	19.51272		-1.854541878674226E-002	
47	19.35376		-1.874630482521099E-002	
48	19.22066		-1.892722217491672E-002	
49	19.07419		-1.911219281743871E-002	
50	18.95207		-1.931828430456439E-002	
51	18.79986		-1.949634468729518E-002	
52	18.67216		-1.966306491643768E-002	
53	18.53104		-1.987703376837287E-002	
54	18.41312		-2.005804010385840E-002	
55	18.28359		-2.025146153291822E-002	
56	18.16457		-2.042669088315748E-002	
57	18.04398		-2.062479540916731E-002	
58	17.93354		-2.079176633512634E-002	
59	17.81902		-2.098584109732824E-002	
60	17.71521		-2.116183822928947E-002	
61	17.60747		-2.135047719887743E-002	
62	17.51089		-2.153182508783954E-002	
63	17.41082		-2.169936172734758E-002	
64	17.32160		-2.185006755155365E-002	
65	17.22170		-2.202332305462285E-002	
66	17.13864		-2.218031880982738E-002	
67	17.05742		-2.232999447470733E-002	
68	16.97846		-2.248882701097013E-002	
69	16.88755		-2.259290531412278E-002	
70	16.80522		-2.270265776503931E-002	

71	16.72525	-2.280469542406496E-002
72	16.64498	-2.292281115087197E-002
73	16.56688	-2.302085936401227E-002
74	16.49288	-2.311976161846199E-002
75	16.42139	-2.323814455001898E-002
76	16.35085	-2.333656636072109E-002
77	16.28152	-2.343536715801672E-002
78	16.21166	-2.351531973205301E-002
79	16.14600	-2.362605659889274E-002
80	16.08357	-2.374048145625374E-002
81	16.01878	-2.383222563799544E-002
82	15.95623	-2.393314702866691E-002
83	15.89571	-2.40676571981253E-002
84	15.83680	-2.417737004145992E-002
85	15.77958	-2.425897567391444E-002
86	15.72440	-2.435703919853898E-002
87	15.64417	-2.448896294933279E-002
88	15.57270	-2.458333781336249E-002
89	15.49617	-2.470573599658040E-002
90	15.42119	-2.477529602900300E-002
91	15.35449	-2.488238792425066E-002
92	15.28288	-2.501107982285475E-002
93	15.22044	-2.511849080237368E-002
94	15.15946	-2.522541290913338E-002
95	15.09961	-2.531567466715599E-002
96	15.04113	-2.542852798802455E-002
97	14.98388	-2.549786324635844E-002
98	14.92779	-2.557659400933895E-002
99	14.86428	-2.566771129622613E-002
100	14.80973	-2.575392003558161E-002
101	14.74867	-2.584019750702430E-002
102	14.69633	-2.588519289899248E-002
103	14.63891	-2.595482131508488E-002
104	14.58803	-2.604027193291135E-002
105	14.53292	-2.612242078174365E-002
106	14.47821	-2.619641851302456E-002
107	14.42389	-2.625011528166922E-002
108	14.36767	-2.633887879637835E-002
109	14.31131	-2.637339141697072E-002
110	14.25455	-2.644290495505031E-002

Table S2. Cartesian coordinates (x,y,z) and partial charges (q) on each atom of bare Hbc^{+} computed using DFT at the M06-2X/6-31+G* level

Atom	*x*	*y*	*z*	*q*
C	1.427694	0.000000	0.002383	-0.067403
C	0.721205	-1.227473	0.003547	0.048757
C	-0.721205	-1.227473	-0.003547	0.042697
C	-1.427694	0.000000	-0.002383	-0.082617
C	-0.721205	1.227473	-0.003547	0.042697
C	0.721205	1.227473	0.003547	0.048757
C	2.870123	0.000000	0.028184	0.016042
C	1.435537	-2.469514	-0.015514	0.003980
C	-1.435537	-2.469514	0.015514	0.030115
C	-2.870123	0.000000	-0.028184	0.068476
C	-1.435537	2.469514	0.015514	0.030115
C	1.435537	2.469514	-0.015514	0.003980
C	4.984395	-1.197447	0.146065	-0.143655
C	2.860433	-2.476789	-0.035041	0.055677
C	0.730751	-3.705819	-0.040789	0.067793
C	-0.730751	-3.705819	0.040789	0.006919
C	-2.860433	-2.476789	0.035041	0.085660
C	-4.984395	-1.197447	-0.146065	-0.113540
C	-4.984395	1.197447	-0.146065	-0.113540
C	-2.860433	2.476789	0.035041	0.085660
C	-0.730751	3.705819	0.040789	0.006919
C	0.730751	3.705819	-0.040789	0.067793
C	2.860433	2.476789	-0.035041	0.055677
C	4.984395	1.197447	0.146065	-0.143655
C	3.583942	-1.222002	0.044938	0.052757
C	1.445769	-4.890617	-0.157635	-0.181232
C	-1.445769	-4.890617	0.157635	-0.160580
C	-3.583942	-1.222002	-0.044938	-0.005018
C	-3.583942	1.222002	-0.044938	-0.005018
C	-1.445769	4.890617	0.157635	-0.160580
C	1.445769	4.890617	-0.157635	-0.181232
C	3.583942	1.222002	0.044938	0.052757
C	3.536758	-3.702472	-0.144961	-0.189055
C	2.839193	-4.888746	-0.215789	-0.056921
C	-2.839193	-4.888746	0.215789	-0.059379
C	-3.536758	-3.702472	0.144961	-0.200515
C	-5.676612	0.000000	-0.206223	-0.123959
C	-3.536758	3.702472	0.144961	-0.200515
C	-2.839193	4.888746	0.215789	-0.059379
C	2.839193	4.888746	-0.215789	-0.056921
C	3.536758	3.702472	-0.144961	-0.189055
C	5.676612	0.000000	0.206223	-0.100632
H	-5.547269	2.119522	-0.201337	0.150474
H	5.547269	-2.119522	0.201337	0.151183
H	4.616101	-3.734044	-0.200691	0.153170
H	-4.616101	-3.734044	0.200691	0.153368
H	-4.616101	3.734044	0.200691	0.153368
H	4.616101	3.734044	-0.200691	0.153170
H	0.933133	-5.840847	-0.226938	0.157573
H	-0.933133	-5.840847	0.226938	0.149897
H	-5.547269	-2.119522	-0.201337	0.150474
H	-0.933133	5.840847	0.226938	0.149897
H	0.933133	5.840847	-0.226938	0.157573
H	5.547269	2.119522	0.201337	0.151183
H	3.373547	-5.826632	-0.319393	0.146595
H	-3.373547	-5.826632	0.319393	0.148317
H	-6.756334	0.000000	-0.301180	0.151021
H	-3.373547	5.826632	0.319393	0.148317
H	3.373547	5.826632	-0.319393	0.146595
H	6.756334	0.000000	0.301180	0.149000

Table S3. Cartesian coordinates (*x*,*y*,*z*) and partial charges (*q*) on each atom of bare Hbc^{-} computed using DFT at the M06-2X/6-31+G* level

Atom	*x*	*y*	*z*	*q*
C	1.425635	0.000000	0.007585	-0.008020
C	0.720650	-1.229313	0.005254	-0.053014
C	-0.720650	-1.229313	-0.005254	-0.052301
C	-1.425635	0.000000	-0.007585	-0.032544
C	-0.720650	1.229313	-0.005254	-0.052301
C	0.720650	1.229313	0.005254	-0.053014
C	2.875561	0.000000	0.037419	-0.017661
C	1.436193	-2.465684	-0.021342	0.021926
C	-1.436193	-2.465684	0.021342	0.046947
C	-2.875561	0.000000	-0.037419	0.047310
C	-1.436193	2.465684	0.021342	0.046947
C	1.436193	2.465684	-0.021342	0.021926
C	4.992916	-1.202170	0.172772	-0.267114
C	2.861685	-2.480965	-0.038745	-0.041590
C	0.733022	-3.708523	-0.052933	0.068006
C	-0.733022	-3.708523	0.052933	0.001256
C	-2.861685	-2.480965	0.038745	-0.005936
C	-4.992916	-1.202170	-0.172772	-0.226459
C	-4.992916	1.202170	-0.172772	-0.226459
C	-2.861685	2.480965	0.038745	-0.005936
C	-0.733022	3.708523	0.052933	0.001256
C	0.733022	3.708523	-0.052933	0.068006
C	2.861685	2.480965	-0.038745	-0.041590
C	4.992916	1.202170	0.172772	-0.267114
C	3.591650	-1.229065	0.057202	0.105057
C	1.446235	-4.893847	-0.193078	-0.220207
C	-1.446235	-4.893847	0.193078	-0.196015
C	-3.591650	-1.229065	-0.057202	0.034750
C	-3.591650	1.229065	-0.057202	0.034750
C	-1.446235	4.893847	0.193078	-0.196015
C	1.446235	4.893847	-0.193078	-0.220207
C	3.591650	1.229065	0.057202	0.105057
C	3.532873	-3.708838	-0.168296	-0.166399
C	2.840612	-4.898010	-0.258953	-0.153656
C	-2.840612	-4.898010	0.258953	-0.159794
C	-3.532873	-3.708838	0.168296	-0.176256
C	-5.676387	0.000000	-0.238748	-0.130540
C	-3.532873	3.708838	0.168296	-0.176256
C	-2.840612	4.898010	0.258953	-0.159794
C	2.840612	4.898010	-0.258953	-0.153656
C	3.532873	3.708838	-0.168296	-0.166399
C	5.676387	0.000000	0.238748	-0.094779
H	-5.553554	2.125551	-0.243011	0.126360
H	5.553554	-2.125551	0.243011	0.127815
H	4.614037	-3.730319	-0.233867	0.116569
H	-4.614037	-3.730319	0.233867	0.115359
H	-4.614037	3.730319	0.233867	0.115359
H	4.614037	3.730319	-0.233867	0.116569
H	0.921357	-5.837712	-0.279613	0.129206
H	-0.921357	-5.837712	0.279613	0.119747
H	-5.553554	-2.125551	-0.243011	0.126360
H	-0.921357	5.837712	0.279613	0.119747
H	0.921357	5.837712	-0.279613	0.129206
H	5.553554	2.125551	0.243011	0.127815
H	3.375397	-5.834287	-0.386429	0.107523
H	-3.375397	-5.834287	0.386429	0.110518
H	-6.757541	0.000000	-0.348535	0.108078
H	-3.375397	5.834287	0.386429	0.110518
H	3.375397	5.834287	-0.386429	0.107523
H	6.757541	0.000000	0.348535	0.103556
